# Ivacaftor affects the susceptibility of standard-of-care drugs used to treat *Mycobacterium abscessus* lung disease

**DOI:** 10.1128/aac.00030-25

**Published:** 2025-05-15

**Authors:** Avneesh Shrivastava, Sanjay Singh, Gunavanthi D. Boorgula, Rodolfo-Amaro Galvez, Shashikant Srivastava

**Affiliations:** 1 Division of Infectious Diseases, Department of Medicine, School of Medicine, University of Texas at Tyler 675071, Tyler, Texas, USA; 2 Department of Medicine, Section of Pulmonary and Critical Care, School of Medicine, University of Texas at Tyler 675071, Tyler, Texas, USA; 3 Department of Cellular and Molecular Biology, University of Texas Health Science Centre at Tyler 12341, Tyler, Texas, USA; City St George's, University of London, London, United Kingdom

**Keywords:** nontuberculous mycobacteria, cystic fibrosis, CFTR modulator, susceptibility

## Abstract

Patients with cystic fibrosis have dysfunctional cystic fibrosis transmembrane conductance regulator (CFTR), and this predisposes them to nontuberculous mycobacteria (NTM), including *Mycobacterium abscessus* (MAB)*,* infection. We found that one of the CFTR modulators, ivacaftor, kills MAB in a concentration-dependent manner, with killing efficacy comparable to amikacin and imipenem, drugs in guideline-based regimens. Using clinical isolates of MAB, amikacin 1/4× MIC concentration combined with ivacaftor killed 2.67 log_10_ CFU/mL MAB.

## INTRODUCTION

The prevalence of nontuberculous mycobacterial (NTM) infection is increasing, including in patients with cystic fibrosis. By one estimate, ~10% of patients with cystic fibrosis in the United States encountered positive NTM cultures at least once (1). NTM infections are difficult to eradicate despite prolonged treatment with multiple intravenous antibiotic regimens (2). One recent meta-analysis reported sustained sputum culture conversion without relapse in only 23% of patients infected with *Mycobacterium abscessus* (MAB) (3).

Patients with cystic fibrosis have dysfunctional cystic fibrosis transmembrane conductance regulator (CFTR), and this predisposes them to NTM infection (4). One retrospective study and a case report that followed a patient for 12 years who was consistently positive for MAB cultures, converted to culture negative after administration of a triple combination elexacaftor/ivacaftor/tezacaftor (5) suggest that treatment with CFTR modulators could be associated with favorable treatment outcomes, including MAB pulmonary disease (6–8). The aim of this study was to provide *in vitro* evidence for the antimycobacterial activity of ivacaftor and its effect on MAB susceptibility to other drugs. We report results from a series of experiments performed with ivacaftor (BOC Sciences, NY, USA) and MAB using the standard reference strain (ATCC 19977) and three clinical isolates randomly selected from a library of 47 species-identified strains.

**Fig 1:**
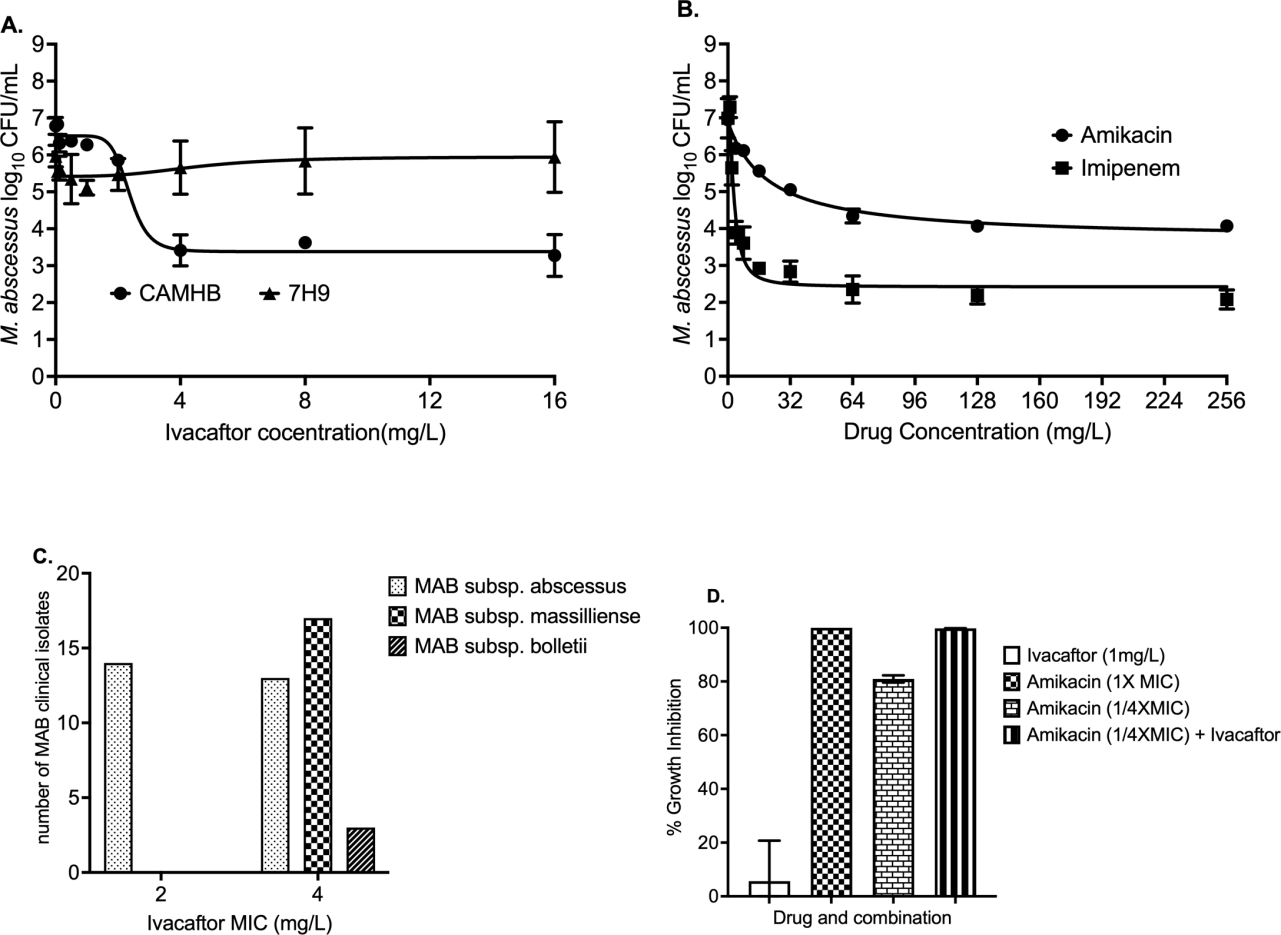
Ivacaftor and *M. abscessus*. (**A**) Ivacaftor did not kill MAB in experiments performed with Middlebrook 7H9 broth, likely due to the high protein binding with albumin in the OADC supplement. However, CAMHB, devoid of any protein in the media, had microbial kill with an extent varying with ivacaftor concentration. (**B**) Amikacin and imipenem concentration-response curves as a comparator to ivacaftor. (**C**) Ivacaftor MIC distribution. For MAB subsp. *abscessus,* MICs ranged between 2 and 4 mg/L. Whereas all isolates in the other two subspecies showed a MIC of 4 mg/L. (**D**) Ivacaftor (1 mg/L) alone did not inhibit MAB growth. While the bacterial burden in cultures treated with amikacin 1/4× MIC concentration was not statistically different from nontreated controls, in combination with ivacaftor killed 2.67 + 0.02 log_10_ CFU/mL MAB.

Since ivacaftor is highly protein-bound, experiments were performed to determine the best medium for drug susceptibility testing and concentration-response studies. Figure 1A shows the results of the study comparing ivacaftor concentration response in Cation adjusted Mueller Hinton broth (CAMHB) and Middlebrook 7H9 with 10% OADC (herein termed “7H9”). We used the inhibitory sigmoid maximal effect model to determine the relationship between drug concentrations and the bacterial burden. In the experiment performed with 7H9, there was no antimicrobial effect in cultures, even with the highest ivacaftor concentration (16 mg/L), and the model failed to fit the data. This lack of ivacaftor efficacy in Middlebrook 7H9 broth could be due to the presence of albumin in the OADC supplement, hence the likelihood of high protein binding reducing free drug concentration to exert an antimicrobial effect. In CAMHB, as shown in Fig. 1A, there was a concentration-dependent microbial killing with ivacaftor. To confirm the antimycobacterial activity of ivacaftor in the CAMHB, the concentration-response study was repeated three times with two replicates per concentration. Figure 1B shows the comparative efficacy of amikacin and imipenem, two drugs included in the guideline-based treatment regimen for MAB (2). Table 1 summarizes the ivacaftor model parameters in comparison with amikacin and imipenem.

**TABLE 1:** Comparison of the inhibitory sigmoid model parameter of ivacaftor with standard-of-care drugs^*a*^^,^^*b*^

	Ivacaftor	Amikacin	Imipenem
Best-fit values			
E_con_ (log_10_ CFU/mL)	6.52 ± 0.10	6.89 ± 0.09	7.31 ± 0.26
E_max_ (log_10_ CFU/mL)	3.14 ± 0.21	3.18 ± 0.19	4.89 ± 0.31
H	7.77 ± 7.96	1.0 ± 0.12	1.77 ± 0.32
EC_50_ (mg/L)	2.37 ± 0.43	20.8 ± 3.02	3.10 ± 0.38
Goodness of fit			
R squared	0.96	0.98	0.94

^*a*^ Data is presented as mean ± standard error.

^*b*^ *E*_con_, bacterial burden in nontreated controls; *E*_max_, maximal kill, which is the difference in the bacterial burden between nontreated controls and the highest drug concentration used in the experiments; EC_50_, drug concentrations to exhibit 50% of the *E*_max_; *H* is the Hill coefficient in the model equation.

Next, we performed Ivacaftor MIC experiments (9) using CAMHB against the MAB standard laboratory strain (ATCC 19977) and a library of 47 species identified MAB clinical isolates (27 MAB subsp. *abscessus*, 17 MAB subsp. *massilliense*, and 3 MAB subsp. *bolletii*). Ivacaftor MIC of MAB ATCC strain was 2 mg/L. For comparison, the amikacin, imipenem, and azithromycin MICs of the ATCC strain were 16, 4, and 64 mg/L, respectively. Figure 1C shows the subspecies-wise MIC distribution among the 47 clinical isolates, where MIC_50_ and MIC_90_ were calculated as 4 mg/L.

Figure 1D shows the results of the experiments performed with ivacaftor (1 mg/L) alone or combined with amikacin 1× and 1/4× MIC concentrations. Ivacaftor alone did not inhibit MAB growth. While the bacterial burden in cultures treated with amikacin 1/4× MIC concentration was not statistically different from nontreated controls, in combination with 1 mg/L ivacaftor, amikacin killed 2.67 + 0.02 log_10_ CFU/mL MAB.

Ivacaftor was developed to treat patients with specific mutations in the CFTR modulator. A recent study analyzed the patient registry from 2011 to 2018, including 25,987 individuals (8). This study reported that 43% of the eligible individuals received CFTR modulator therapy, and 23% had NTM infection (8). There was no significant difference in the NTM culture positivity hazard between individuals receiving ivacaftor monotherapy versus triple combination therapy of elexacaftor/ivacaftor/tezacaftor. There are also a few other reports of reduction in the number of NTM isolations in patient samples after initiation of triple combination elexacaftor/ivacaftor/tezacaftor (7) as well as the potential antimicrobial effect of ivacaftor in patients with cystic fibrosis (10, 11). However, the mechanism of the antibacterial properties of ivacaftor that structurally resemble quinolones is unknown (10). Elsewhere, structural modifications in quinoline derivatives have been reported to affect the antibacterial activity and pharmacokinetics of the drugs (12).

In the present study, we performed drug-susceptibility testing experiments with three randomly selected MAB clinical isolates using three different ivacaftor concentrations (0.5, 1.0, and 2.0 mg/L) to determine if ivacaftor can reduce the MIC of amikacin and azithromycin when used in combination (Table 2). Indeed, even the lowest ivacaftor concentration (0.5 mg/L) was able to reduce the amikacin and azithromycin MICs by multiple folds. For a clinical context, ivacaftor 150 mg has been found to achieve peak concentrations of 1.15 mg/L in patients aged between 12 years and <18 years, and 1.27 mg/L in patients >18 years of age (5). Ivacaftor exposures have also been found to increase by 2.5- to 4-fold when the drug is administered with fat-containing meals (5).

**TABLE 2:** Shift in amikacin and azithromycin minimum inhibitory concentration in the presence of ivacaftor^*a*^

Drug	Ivacaftor (mg/L)	Isolate_1	Isolate_2	Isolate_3
Amikacin	0	16	16	32
	0.5	4	4	4
	1	4	4	4
	2	4	2	2
Azithromycin	0	>64	64	64
	0.5	32	16	16
	1	32	8	8
	2	32	8	8

^*a*^ The combination of 0.5 mg/L (1/4× MIC) ivacaftor resulted in multiple-fold lower amikacin and azithromycin MICs.

In summary, ivacaftor alone killed MAB, reduced the MIC of amikacin and azithromycin in combination, and showed comparable antimycobacterial activity to several drugs included in the MAB clinical practice guidelines (2). Further preclinical and clinical studies are warranted with this more tolerable oral drug to optimize the MAB treatment regimens.

## Data Availability

The raw data for the results presented in the manuscript is available from the corresponding author, upon a reasonable request following UTHSCT’s data-sharing policy.
